# Withdrawn as Duplicate: Public Health competences: Prioritisation and leadership

**DOI:** 10.1093/eurpub/ckw127

**Published:** 2022-06-01

This paper was published twice due to an administrative error. The correct version can be found at https://doi.org/10.1093/eurpub/ckw125

